# New stably transfected bioluminescent cells expressing FLAG epitope-tagged estrogen receptors to study their chromatin recruitment

**DOI:** 10.1186/1472-6750-9-77

**Published:** 2009-09-09

**Authors:** Eric Badia, Aurélie Escande, Patrick Balaguer, Raphaël Métivier, Vincent Cavailles

**Affiliations:** 1 IRCM, Institut de Recherche en Cancérologie de Montpellier, F-34298; INSERM, U896, F-34298; Université Montpellier1, F-34298; CRLC Val d'Aurelle Paul Lamarque, Montpellier, F-34298, France; 2 Université de Rennes I, CNRS, UMR 6026, Equipe SPARTE, IFR140 GFAS, Campus de Beaulieu, 35042 Rennes cedex, France

## Abstract

**Background:**

Biological actions of estrogens are mediated by the two specific estrogen receptors ERα and ERβ. However, due to the absence of adequate cellular models, their respective transcriptional activities are still poorly understood. For instance, the evaluation of such differing properties on the transcription of responsive genes using ChIP experiments was hindered by the deficiency of cells exhibiting the same genotypic background and properties but expressing only one of the ERs. We describe here the generation of such cells, using an estrogen receptor negative HELN cell line that was derived from HeLa cells stably transfected with an ERE-driven luciferase plasmid. These HELN-Fα and HELN-Fβ cell lines stably express either the alpha or beta (full length) estrogen receptor tagged with the FLAG epitope. The use of antibodies directed against the FLAG epitope allowed a direct comparative evaluation of the respective actions of both ERs using ChIP.

**Results:**

HELN-Fα and HELN-Fβ cell lines were found to express comparable levels of their corresponding tagged receptors with a Kd for estradiol binding of 0.03 and 0.27 nM respectively. The presence of a stably transfected ERE-driven luciferase plasmid in these cells allowed the direct evaluation of the transcriptional activity of both tagged receptors, using natural or synthetic estrogens. FLAG-ERα and FLAG-ERβ were found to exhibit similar transcriptional activity, as indicated by a kinetic evaluation of the transcriptional activation of the luciferase gene during 10 hrs of treatment with estradiol. The validity of these model cells was further confirmed by the predictable transcriptional regulations measured upon treatments with ERα or ERβ specific ligands. The similar immunoprecipitation efficiency of both tagged receptors by an anti-FLAG antibody allowed the assessment of their kinetic recruitment on the synthetic luciferase promoter (containing an estrogen response element) by ChIP assays during 8 hours. A biphasic curve was obtained for both FLAG-ERα and FLAG-ERβ, with a peak occurring either at 2 hr or at 1 hr, respectively, and a second one following 4 hr of E2 stimulation in both cases. In MCF-7 cells, the recruitment of ERα also exhibited a biphasic behaviour; with the second peak however not so important than in the HeLa cell lines.

**Conclusion:**

In HELN derived cell lines, no fundamental differences between kinetics were observed during 8 hours for FLAG-ERα and FLAG-ERβ, as well as for polymerase II recruitment. However, the relative importance of recruitment between 1 hr and 4 hr was found to be different in HeLa cell line expressing exogenous tagged ERα and in MCF-7 cell line expressing endogenous ER.

## Background

Estrogen receptors (ERα and ERβ) are ligand-activated transcription factors encoded by two different genes, located respectively on chromosome 6 and 14 [1,2]. Since the discovery of ERβ [3], numerous studies tried to clarify its biological roles in addition to those mediated by ERα [4-6]. Nevertheless, the complex interplays between the two receptors are far from being fully understood. The specific tissue and cell distribution of each receptor is a primary level of such complexity; some tissues preferentially expressing one type of receptor (for instance, ERα was primarily found in the uterus and ERβ in the ovary), and others like breast expressing both receptors [7].

Secondly, both receptors are expressed in several isoforms, and especially the ERβ for which at least 6 isoforms have been found [8-11]. The original ERβ clone encoded a protein of 485 amino acids (ERβ1 short) and later on, a longest form of 530 amino-acids (ERβ1 long) has been cloned and is currently considered as the full-length wild-type ERβ [12]. The analysis of transgenic mice lacking either ERα or ERβ or both receptors provided a picture of the specific roles of these receptors. The prominent role of ERα was confirmed in most classic estrogen target tissues, including the mouse mammary gland during development, whereas ERβ was found to achieve the terminal differentiation of this organ [13]. A role for ERβ has also been found in ovary and cardiovascular system [5].

A third level of complexity is linked to the subtly differing transcriptional potentialities of these receptors. Some of these dissimilarities may obviously be linked to the respective intracellular level of each receptor, but also to their ability to heterodimerize and to interact with the complex battery of transcriptional coregulators. In many instances, ERβ seems to oppose the actions of ERα, as it is for instance exerting a concentration dependent reduction of ERα transcriptional potency [14]. A precise analysis of the mechanisms involved in transcriptional regulation in intact cells has become possible with the emergence of the chromatin immunoprecipitation (ChIP) approach, which was used by several kinetic studies of nuclear receptor recruitment to their response elements, along with the myriad of associated coregulators [15]. Some studies, focused on ERα and performed on short time scales of hormone treatment, demonstrated a cyclic recruitment of the receptor and some associated cofactors on the estrogen-responsive elements (EREs) from the pS2 or cathepsin D genes [15-19]. Later on, it was shown that cyclic methylation of the pS2 promoter was also associated with the cyclical recruitment of ERα and cofactors, making the picture of transcriptional events far more complex than imagined [20].

Concerning the study of the recruitment of ERβ on DNA, the existence of both various ER(+) and ER(-) cellular models and receptor isoforms [21] added a combinatorial complexity for determining experimental strategies. Except the personal "LBD" antibody used by Liu [22], most currently available antibodies for ERβ did not appear suitable for ChIP assays. So far, few cellular models expressing ERα and ERβ alone or in combination have been described. A first series of cellular models has been engineered by transfecting the ERα positive MCF-7 cell line with a plasmid expressing an inducible FLAG-tagged version of ERβ. Such MCF-7 based models have been used by Matthews to study the recruitment of ERα in the presence or absence of the short ERβ1 isoform [23], by Liu for Chip-on-chip analysis of ERα- and ERβ-binding DNA regions [22], and by Murphy for transcriptional and growth responses analysis [24]. A second kind of model has been engineered by transfecting the ER negative breast cancer Hs578T cell line with a FLAG-tagged and inducible version of ERα, ERβ or ERβ cx, and was used for microarray analysis of gene expression [25].

In the present study, we generated two independent cell lines (HELN-Fα and HELN-F β) by introducing FLAG versions (stable transfection) of either ERα or ERβ (long form: 530 amino acids) in an ER negative HeLa cell line previously stably transfected with an ERE-driven luciferase reporter gene. These cellular models combine the ease of bioluminescence analysis of estrogen inducible reporter transgene transcription with the power of ChIP analysis of both receptors (ERα and β) recruitment in an identical cell context and with the same antibody.

## Results

### Characterization of HELN-Fα and HELN-Fβ cell lines

#### Expression and binding properties of tagged receptors

Generation of HELN-FLAG-ERα (HELN-Fα) and HELN-FLAG-ERβ (HELN-Fβ) cell lines was performed in two steps. An estrogen-responsive reporter gene in which luciferase expression is driven by an estrogen-responsive element (ERE) in front of the βGlobin promoter (ERE-βGlobin-Luc-SVNeo) was first stably introduced into human cervix adenocarcinoma cells (HeLa) cells generating the HELN cell line [26]. In a second step, HELN cells were stably transfected with plasmids encoding human FLAG-ERα or FLAG-ERβ (full length version of 530 amino acids) to obtain HELN-Fα and HELN-Fβ cell lines, respectively.

**Figure 1:**
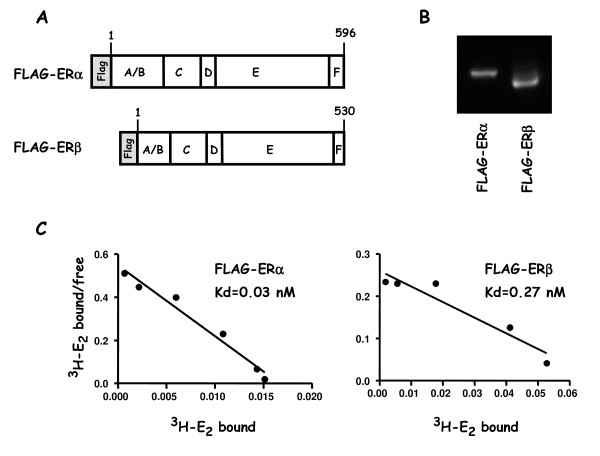
**Structure, expression and binding properties of the FLAG-ERα/β proteins**. **A) **Representation of FLAG-ERα/β proteins showing the amino acid sequence length of ERα and ERβ, as well as their different domains, and the position of the FLAG tag. **B) **Western blot using the M2 anti-FLAG antibody to probe the relative amounts of FLAG-ERα or FLAG-ERβ present in 40 μg of total protein extracts prepared from HELN-Fα or HELN-Fβ stable cell lines cultured in 3% DCC (in absence of phenol red). **C) **Scatchard plot analysis of specific binding of [3H]-E2 to FLAG-ERα or FLAG-ERβ during 6 hours in a whole cell assay. Dissociation constant (Kd) is indicated.

The 3× FLAG epitope (FLAG) fused to the N-terminus of the receptors (Figure 1A) was used to analyse their expression by Western blotting in the different clones of HELN-Fα and HELN-Fβ obtained. Two cell lines that displayed comparable levels of FLAG-ERα and FLAG-ERβ (Figure 1B) were selected and then used for all following experiments. FLAG-ERα and FLAG-ERβ receptor protein levels, as well as their respective affinities for estradiol, were more precisely determined using ^3^H-estradiol in saturation experiments (Figure 1C). Scatchard analysis performed in whole cell assays, led to a protein determination of 50 fmoles/mg and 159 fmol/mg and a dissociation constant of 0.03 nM and 0.27 nM for FLAG-ERα and FLAG-ERβ respectively. These dissociation constant values were similar to those previously found for untagged receptors [27]. Indeed, in HELN-ERα and HELN-ERβ cell lines, Kd were 0.04 and 0.11 nM respectively thus indicating that the FLAG tag do not interfere with estradiol binding. In the parental HELN cell line, ERs (α or β) were not detected by binding experiments [27].

#### Transactivation analysis of tagged receptors

**Figure 2:**
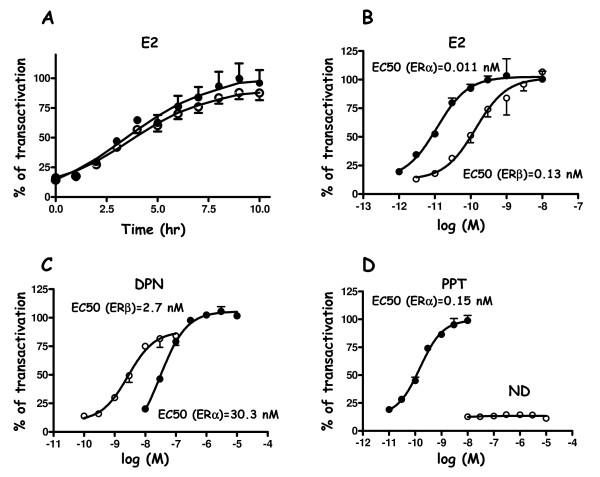
**Kinetic analysis of luciferase induced expression in HELN-Fα/β cells and effect of selective agonist ligands on transcriptional activity**. **A) **Kinetic analysis of luciferase expression of HELN-Fα and HELN-Fβ stable cell lines stimulated by 10 nM E2 during 10 hours. Luciferase activity was then recorded every hour and expressed as the percentage of maximal activity. HELN-Fα or HELN-Fβ stable cell lines were stimulated 16 hr with either E2 **(B)**, DPN **(C) **PPT **(D) **at indicated concentrations. Maximal activities (100%) correspond to the activity obtained with a 10 nM E2 stimulation. Values are mean ± S.D. from quadruplicate experiments. In **A, B, C, D**, filled circle were for HELN-Fα and empty circles for HELN-Fβ.

A comparative study of the transactivation properties of each of the tagged receptors was next undertaken by analysing the luciferase activity of HELN-Fα and HELN-Fβ cell lines upon estradiol treatments. Firstly, we performed a kinetic analysis of ERE-driven luciferase gene induction during prolonged estradiol stimulation (10 hr), which is an agonist ligand for both receptors. As shown in Figure 2A, a significant and similar induction of luciferase activity was detected in both cell lines containing the respective tagged version of each receptor. Luciferase activities expressed per mg of protein also confirm the homogeneity of the hormonal response obtained in these two cell lines (data not shown).

We next determined EC_50 _values for estradiol and various agonist selective synthetic ligands. Propylpyrazoletriol (PPT) has been described in the literature as an ERα selective agonist with a relative binding activity (RBA) of 49, (as compared to an RBA = 100 for estradiol), whereas the diarylpropionitrile compound (DPN) displays ERβ agonist selectivity (RBA = 18) [28,29]. Concerning E2 dose response curves (Figure 2B), we found an EC_50 _for ERβ (0.13 nM) higher than for ERα (0.011 nM). As expected, DPN showed a selective activation of ERβ when used at a concentration less than 10 nM, with an EC_50 _of 2.7 nM (Figure 2C) and PPT activated only ERα (EC50 = 0.15 nM) (Figure 2D). These results are consistent with those obtained in our laboratory using HELN cells transfected with untagged version of ERα or ERβ [27]. Collectively, these results showed that HELN-Fα and HELN-Fβ cell lines exhibit similar features than HELN cell lines containing the corresponding untagged version of the receptors [27].

### ChIP analysis of ERα, ERβ kinetics recruitment during sustained E2 stimulation

The main objective of this work was to create cellular models allowing a facilitated comparative analysis of some cellular properties of ERα and ERβ, when placed in an identical context. The results described above suggested that these models were relevant and could be useful to further compare hormone-dependent chromatin mediated recruitment of these receptors.

#### Preliminary experiments

**Figure 3:**
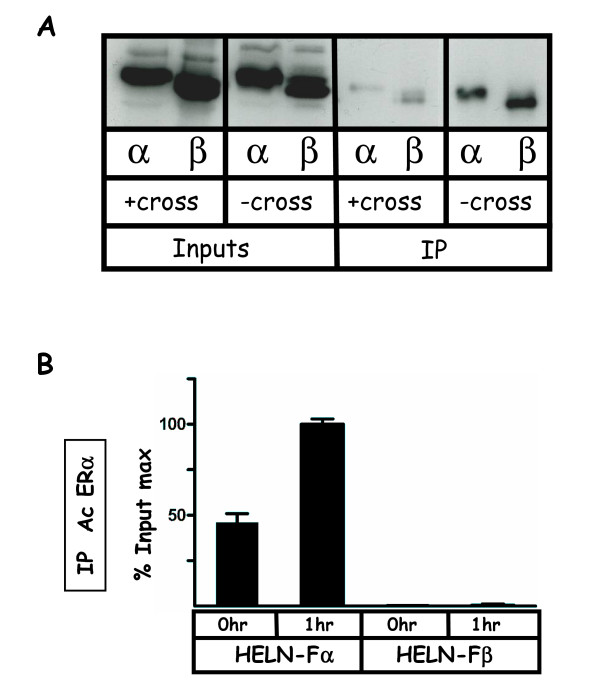
**Compared efficiencies of FLAG-ERα/β immunoprecipitations**. **A) **Soluble chromatin was prepared from HELN-Fα or HELN-Fβ cells stimulated 1 hr with 10 nM of E2, either by using chromatin cross-linked with the same procedure as that used for kinetic ChIP experiments (named "+cross"), or by using uncrosslinked chromatin solubilized in a mild buffer (named "-cross"). Then, 40 μg of ± cross-linked chromatin were subjected to a 10% slab gel electrophoresis before (Inputs) or after (IP) immunoprecipitation with the anti-FLAG antibody. **B) **HELN-Fα or HELN-Fβ cells were stimulated 1 hr with 10 nM of E2, then, soluble chromatin preparation and immunoprecipitation (with the HC20 anti ERα antibody) were performed by using an identical procedure than that used for ChIP-kinetics experiments. Real time PCR quantification of either IP chromatin or input was performed at each incubation time. Amplified signals from IP chromatin were calculated as the percentage of amplified input signals obtained during the same amplification. Corresponding values plotted at indicated time were expressed as a percentage of the maximum value obtained in this experiment. Values are mean ± SD of two independent immunoprecipitation assays.

We first verified that the efficiencies of immunoprecipitations (IPs) of FLAG-ERα or FLAG-ERβ proteins using the anti-FLAG antibody were comparable. As shown in Figure 3A, signals obtained after Western blotting using extracts from HELN-Fα or HELN-Fβ cell lines were of similar amplitude when IPs were done either in absence or presence of the formaldehyde crosslink step which is required for ChIP assays. It should be noted that the immunoprécipitations using cross-linked chromatin had a lower efficiency probably due to the worse accessibility of the FLAG epitope in these conditions.

ChIP specificity was then addressed using extracts from HELN-Fα and HELN-Fβ cells that were cross-linked with formaldehyde (as described in methods), either at time zero or after 1 hr of E2 treatment. ChIPs were performed using the specific anti-ERα HC-20 antibody (Santa Cruz Biotechnology) and PCR amplifications with a set of specific oligonucleotides corresponding to a region surrounding the ERE driving luciferase expression. As expected, DNA amplification occurred only on chromatin originating from HELN-Fα cells and not at all with that from HELN-Fβ (Figure 3B). When amplifications were realised with specific oligonucleotides corresponding to the estrogen-inducible pS2 promoter (which is the most studied in the literature), no signal was observed in both HELN-Fα and HELN-Fβ although it was apparent in MCF-7 cells, indicating that this promoter could not be used in these cells for such experiments (data not shown).

#### ChIP kinetic experiments

**Figure 4:**
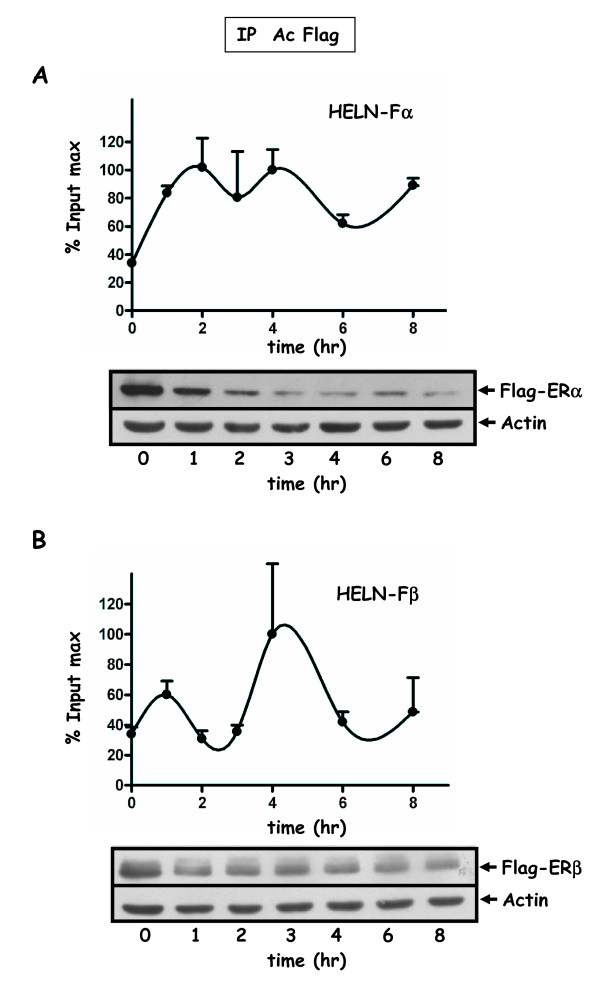
**Recruitment of ERα/β to the promoter of the luciferase transgene in HELN-Fα/β cells**. Kinetic ChIP experiments were performed using the anti-FLAG antibody. HELN-Fα (**A**) or HELN-Fβ (**B**) cells were cultured in 3% dextran-charcoal treated FCS. Twenty four hours before experiment, they were deprived of serum and subsequently treated for 2 hr with 2.5 μM α-amanitin, and then with 10 nM E2. Cells were cross-linked at indicated times. Soluble chromatin was prepared on sampled cells at indicated times as described in material and methods. Real time PCR quantification of either IP chromatin or input was performed at each incubation time. Amplified signals from IP chromatin were calculated as the percentage of amplified input signals obtained during the same amplification. Corresponding values plotted for one curve were expressed as the percentage of the maximum value of % input (% input max) obtained for that curve and for one IP. One ChIP kinetic curve shown is representative of at least two independent experiments and values are mean ± SD of two independent immunoprecipitation assays using the same preparation of chromatin. The % input max average value (corresponding to the two IPs) of one curve may fluctuate among different independent experiments corresponding to the same kinetic. The corresponding amplitudes of variation are: in **A **(HELN-Fα) Imin = 2.1, Imax = 4.3; in **B **(HELNβ) Imin = 1.4 Imax = 3.5. Flag-ERα (A) or Flag-ERβ (B) protein levels were analyzed by Western blot experiments. Cell treatments for ChiP assays and Western blot experiments were identical. Extracts were prepared at indicated times, and Western blotted with antibodies for FLAG (F3165) or actin.

**Figure 5:**
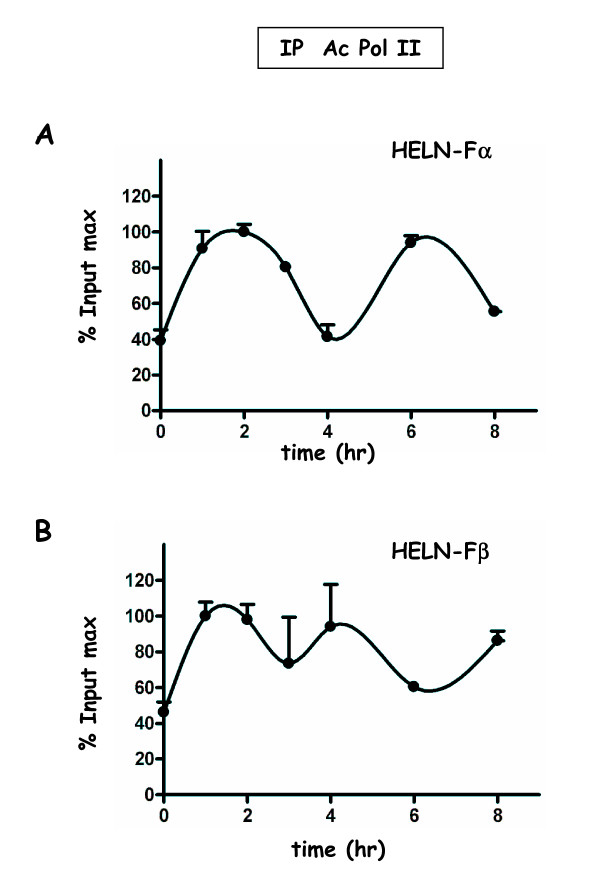
**Recruitment of Pol II to the promoter of the luciferase transgene in HELN-Fα/β cells**. Kinetic ChIP experiments were performed using anti-Pol II antibody. Chromatin samples were obtained and data processed as described in Figure 4. The corresponding amplitudes of variation are: in **A **(HELN-Fα) Imin = 1.5, Imax = 3.1; in **B **(HELNβ) Imin = 2.5 Imax = 3.0.

The ultimate aim of these experiments was to analyse the respective characteristics of ERα and ERβ recruitment on the estrogen inducible stably transfected luciferase transgene within an identical cell context. The time scale that has already been studied with great details in the literature concerned the recruitment of ERα on the pS2 promoter during the first 180 minutes of stimulation [19]. In our study, we have chosen to focus on events that operate on a longer time scale (0-8 hr) on the luciferase transgene promoter. Transcription of cells was first reset by an α-amanitin treatment in order to synchronize and maximize the signal obtained after E2 stimulation. In fact, the effect of α-amanitin treatment is probably more important during the first two hours of estradiol stimulation, and has been used for the detailed analysis of cyclical recruitment of ERα in MCF-7 cells during this time scale [19]. After two hours of α-amanitin treatment, cells were treated with 10 nM of E2 during 8 hours, and cross-linked at indicated times (see methods and figure legend). One difficulty that was encountered was the strict reproducibility of the range of the % input scale when two independent kinetics of recruitment were compared, even though curves shapes were comparable. This can reveal subtle differences in the cell population which may occur among cell cultures (cell passages, cell density...). One representative kinetic ChIP experiment from two independent IPs performed on the same chromatin is shown (Figure 4 and 5). The range of variation of the maximum average value of one curve for all kinetics is indicated (see figure legend).

**Figure 6:**
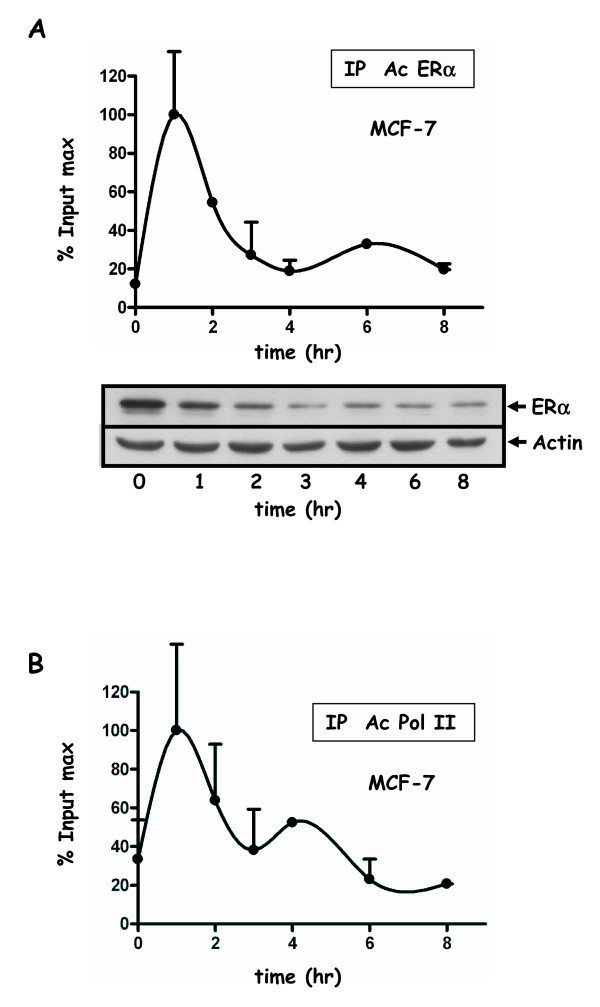
**Recruitment of ERα and Pol II to the pS2 promoter in MCF-7 cells**. ChIP-kinetic experiments were performed using anti ERα antibody (HC-20) (**A**) or anti-Pol II antibody (**B**). Cells were cultured in 10% FCS and then in 3% dextran-charcoal treated FCS for three days. Twenty four hours before experiment, cells were deprived of serum and subsequently treated for 2 hr with 2.5 μM α-amanitin, and then with 10 nM E2 during 8 hours. Chromatin was prepared as in Figure 4 or 5. Values are mean ± SD of two independent immunoprecipitation assays of the same chromatin, and expressed as in Figure 4. ERα (A) protein levels were analyzed by Western blot experiments. MCF-7 treatments for ChiP assays and Western blot experiments were identical. Extracts were prepared at indicated times, and Western blotted with antibodies for ERα (HC-20) or actin.

The common and reproducible feature emerging from these experiments is the biphasic character of the curves that was observed for either FLAG-ERα or FLAG-ERβ recruitment in HELN derived cells, as well as for untagged ERα in MCF-7 cells (Figure 4, 5 and 6). A first peak occurs between 1 and 2 hr of E2 stimulation, and a second one around the fourth hour. The curve shape suggests that a third peak might occur around the ninth hour if the treatment had been extended. Interestingly, we found that when observed with a greater time scale than what was previously studied [19], the recruitment of both receptors still appeared cyclical. Such biphasic curves were also observed for the recruitment of the RNA-polymerase II (Pol II).

Protein levels of either the untagged or tagged estrogen receptor were analyzed by Western blot experiments in MCF-7 or HELN-derived cell lines respectively (Figure 4 and 6). Interestingly, for ERα (tagged or not), the protein level decreased during the first three hours and then remained stable until the eighth hour (Figure 4A). For FLAG-ERβ, the protein level also decreased but with a lower amplitude and an earlier stabilization (Figure 4B). No correlation was found between the level of the FLAG-ERα or FLAG-ERβ receptor protein and the efficiency of their recruitment on the luciferase transgene promoter in HELN cells. However, in MCF-7 cells, the level of the endogenous ERα receptor recruitment on the pS2 promoter was low between the fourth and eighth hour of estradiol treatment which was precisely the time period that correspond to a low estradiol receptor level (Figure 6).

Apart from a shift concerning the second peak of FLAG-ERα (Figure 5), the recruitment of Pol II and FLAG-ERs were almost concomitant. Biphasic shapes were also observed when ERα and Pol II recruitments were analyzed on the pS2 gene promoter in MCF-7 cells (Figure 6). However, in that case, the recruitment of ERα as well as that of Pol II was predominant during the first 2 hours of estradiol treatment.

## Discussion

During the past few years, a highly complex picture of hormone-regulated transcription has begun to emerge from the literature [15,30]. Fluorescence recovery after photobleaching (FRAP) as well as chromatin immunoprecipitation (ChIP) technologies have allowed a rapid evolution of knowledge. Gene transcription now appear to be a very dynamic process that involves a complex interplay between transcription factors, their associated cofactors and chromatin [15]. Concerning estrogen receptors, ChIP studies have begun to address this complex topic by analyzing recruitment kinetics. A pioneering study performed by Shang and collaborators [17] has specified the time course of ERα and associated cofactors interaction on the cathepsin D gene ERE in MCF-7 cells. In that case, one peak occurred at 45 min and a second one at 135 min of E2 stimulation. The recruitment of ERα was also analyzed on the pS2 gene promoter in MCF-7 cells [16]. In this work, three peaks were detected, one at 30 min, a second at 75 min and the last at 135 min after E2 stimulation. However, in a similar study on the pS2 gene promoter in MCF-7 cells, Subramanian and collaborators showed that a sustained recruitment was maintained from 30 min to 90 min [31]. In all the above-mentioned studies, the use of α-amanitin to reset the transcription was not mentioned. Two major studies performed by Métivier and collaborators led to a more precise picture of ERα recruitment on the pS2 promoter along with a myriad of cofactors, either in MCF-7 [19] or in MDA:hERα cells [20]. In these studies, the dynamics of associated cofactors recruitment as well as of CpG methylation were characterized in detail [20,32]. In MCF-7 cells, ERα exhibited a cyclical recruitment with an unproductive peak at 20 min, and a second productive one at 60 min and then at 100 and 140 min, with determinations done every 5 minutes. In ER-negative MDA-MB231 cells (MDA::hERα), ERα introduction led to recruitment cycles that mirror those observed in ER-positive MCF-7 cells [20]. By contrast, in our HELN-Fα or HELN-Fβ cell lines, we found that ERα was not significantly recruited to the pS2 gene promoter. Finally, a recent study described the recruitment of ERα on the ERRα gene promoter with a major peak was observed at 30 min of stimulation and a second one at 120 min [33].

In the present study, we used the promoter of the luciferase transgene to compare the recruitment of FLAG-ERα and FLAG-ERβ. Some discrepancies, both in the number and the time of appearance of recruitment peaks were apparent in the literature cited above, which probably reflect the complexity of all the parameters that could influence the time course of binding to chromatin such as differences between promoters, parameters of cell culture, cell passages, cell densities, pre-treatment or not with α-amanitin, origin of the cell line, mycoplasma contaminations etc... Despite these variable parameters, the differences that were found between the recruitment kinetics of FLAG-ERα and FLAG-ERβ were relatively modest, all experiments showing a biphasic interaction with the maximum percent of input for the two tagged receptors included in similar ranges. One difficulty to fully interpret the data during long-term stimulations is the fact that the curves we obtained might correspond only to a few points of a curve that have in reality a greater oscillation frequency, the former one being an envelope curve of the latter. However, synchronism differences between the transcription effects of both tagged receptors could be attained by our experimental procedure.

An interesting although still debated question is the possible relationship between ERα expression level and the efficiency of both its recruitment on promoters and the resulting transcription. In our study, after amanitin treatment of MCF-7 cells, the global ERα decrease was concomitant with a reduction of the recruitment of the receptor (Figure 6). However, this was not the case for FLAG-ERα,β receptors in HELN cell lines (Figure 4), yet the FLAG-ERα protein level was 50 fmol/mg in HELN-Fα and was similar to the endogenous ERα protein level (98 fmol/mg) in a MCF-7 derived cell line that was generated from our MCF-7 (personal communication from P Balaguer). The very similar down-regulation observed after estradiol stimulation of both FLAG-ER-α and ERα in HELN-Fα and MCF-7 cell lines respectively, could not explain the differences observed for the second peak (4 hr) of the receptor recruitment curve (Figure 4A and Figure 6A). It has been suggested that proteasome-mediated degradation is required for ERα-mediated transcription. For instance, in HeLa cells, the MG132-mediated stabilization and up-regulation of transiently transfected ERα lead to attenuation of E2-responsive gene expression [34]. Moreover, it was shown that a high level of tetracycline inducible estrogen receptor in MCF-7 cells could activate transcription through non canonical mechanisms [35]. Furthermore, in the study of Park et al, the stabilization and up-regulation of ERα by Akt was accompanied by a simultaneous reduction in its transcriptional activity [36]. On the other hand, Reid et al demonstrated that in MCF-7 cells, ERα-mediated cycling on the pS2 promoter was dependent upon the efficiency of the proteasome-induced degradation process [18]. However in that case, the level of total immunoprecipitated ERα was found unchanged during the time course of the experiment (120-180 min), suggesting that the global level of the receptor was not the driving force for this phenomenon. Conversely, in our laboratory, we observed a dissociation of stress-inducing agents on the accumulation of ERα and on its transactivation [37]. These data suggest that other parameters than simply the ERα protein level are required to clarify links that exist between ER processing and transactivation efficiency or receptor recruitment. As mentioned above, accumulation of estrogen receptor protein (induced by MG132 or Akt) can lead, depending on the context, to a decrease of transactivation, but on the contrary, the threshold under which the ER protein level reduction could alter its recruitment to promoters is not clearly defined and might be dependent upon the cellular context.

Another point of this study concerned the position of the Pol II recruitment peaks relative to those of ERs. More particularly, the second Pol II recruitment peak (maximum at 6 hr) was delayed compared to the one of ERα (maximum at 4 hr) in HELN-Fα but not in the HELN-Fβ cells. One can hypothesize that when the time period analyzed is very close from the time zero of α-amanitin release (during the first 2 hours of estradiol treatment), the binding of both Pol II and ER (α or β) is practically concomitant in all cell types, whereas when the time period analyzed is more distant, this synchronization is abolished in a cell specific manner. In addition, the decrease of the receptor protein level might also influence the lost of synchronization for the binding of these factors.

In a study following the influence of ERβ on ERα transcription, a model of estrogen positive T47D cell line containing a tet-off inducible FLAG-ERβ (corresponding to the short ERβ1 isoform) was used [23]. One cannot in principle, exclude the possibility that in such model, the presence of endogenous ERα can alter the kinetic of FLAG-ERβ recruitment. However, ChIP kinetics performed during 150 minutes, showed one recruitment peak of FLAG-ERβ on pS2 and PR gene promoters, between 60-75 minutes. This time scale is in agreement with the first peak we observed in our HELN-Fβ cell line. When ERβ was not expressed, only one recruitment peak of endogenous ERα lasting from 60-75 min of E2 stimulation was detected, this kinetic profile being not significantly affected when FLAG-ERβ was coexpressed [23]. According to the authors, no oscillatory recruitment was observed for either receptor subtype whose kinetics of recruitment were similar during the time course analyzed.

Three other expression models for ERβ have been created, but were not used for ChIP kinetics experiments: a first one was a stable MCF-7 expressing tet-off inducible FLAG-ERβ2 (also termed ERβ cx) as well as stable HEK293 expressing Tet-on inducible FLAG-ERβ2 [38], the other by Hodges-Gallagher being a stable MCF-7 expressing Tet-off inducible ERβ full length (530AA) [39]. The comparison of ChIP kinetics in these different model systems would be informative concerning the links between cellular context and ER transcriptional competence. In our study, the comparison of the kinetics measured in MCF-7 or HELN-Fα/β (Figure 4 and 6) evidences a difference in the amplitude of the second recruitment peak for the receptors as well as for Pol II. One can hypothesize that such differences might affect responses during sustained stimulations.

## Conclusion

In conclusion, HELN-Fα/β cells are proven here to be useful models to analyse the recruitment of tagged version of ERα and ERβ through the ChIP approach. The measurement of cell luminescence allowed a rapid and easy determination of the transactivation properties of the tagged receptors. In these models, the kinetics of recruitment of FLAG-ERα and FLAG-ERβ did not reveal major differences when analyzed during 8 hours. However, although the biphasic shape of the curve was a constant feature, the relative maximum value of both peaks were different when ERα recruitment was compared between HeLa and MCF-7 cell lines, suggesting a role for cell specificity in the process. More work could be done with these cellular models in order to refine the sophisticated picture of transcription that has begun to emerge. In particular, the kinetic studies of cofactor recruitment by each tagged receptor together with the comparison of full and partial agonist ligands should provide new interesting data.

## Methods

### Materials

Materials for cell culture came from Life Technologies (Cergy-pontoise, France). Luciferin (sodium salt) was purchased from Promega (Charbonières, France). 17β-estradiol (E2) was from Sigma-Aldrich (Saint-Quentin Fallavier, France), 4-hydroxytamoxifen was from Zeneca (Macclesfield, UK). DPN and PPT were from Tocris (Ellisville, MO). Anti ERα (HC-20; sc-543) and anti Pol II (N-20; sc-899) antibodies were from Santa-Cruz Biotechnology. Anti-FLAG M2 agarose was from Sigma-Aldrich. Protein A sepharose CL4B was from GE-Healthcare Bio-Sciences AB. DMEM-F12 (21041) was from Invitrogen (France). Monoclonal M2 Anti-FLAG antibody (F3165) was from Sigma (France).

### Plasmid construction

A 3× FLAG sequence was inserted in front of the ERα or ERβ full-length (530 amino acids) sequence by using a set of oligonucleotides whose sequence was in between the 3× FLAG and the 5' part of ERα or ERβ. For 3× FLAG-ERα construct, a first set of oligonucleotides was used for 3× FLAG amplification:

(S.1) ACCT**GGATCC**GCCGCCACCATGGACTACAAAGACCATGAC;

(AS.1) gactggtaccgatatcagatcTATCGATGA. A second set of oligonucleotides was used to amplify ERα sequence: (S.2)GATCTGATATCGGTACCAGTCaccatgaccctccacaccaaagc

(AS.2) ATACGC**GGATCC**TCAGACTGTGGCAGGGAAACCCTC (part in bold: BamH1 sites, part in small: overlapping parts). A second round of PCR was done by using S.1+AS.2, and the resulting PCR containing the fused sequences was cloned in the BamH1 site of a pSG5-puro plasmid. The 3× FLAG-ERβ construct was done by using the same procedure with the following oligonucleotides: S.1 and AS.1 were identical.

(S.2) was GATCTGATATCGGTACCAGTCgatataaaaaactcaccatctag;

(AS.2) was ATACGC**GGATCC**TCACTGAGACTGTGGGTTCTGGG. Expression of receptors is under the control of a CMV promoter.

### Cell culture

HELN-Fα/β cells were routinely grown in DMEM-F12 (phenol red free) supplemented with 3% of dextran-coated charcoal-treated fetal calf serum (DCC medium) and serum was deprived 24 hr before kinetics ChIP analysis. MCF-7 cells were routinely grown in DMEM-F12 containing phenol red supplemented with 10% fetal calf serum (FCS medium). Before ChIP-kinetic analysis, cells were placed in DCC medium during three days, then serum deprived during 24 hr.

### ERα and ERβ transactivation assays

HELN-Fα/β cells were seeded at a density of about 4 × 10^4 ^cells/well in 96-well white opaque tissue culture plates (Greiner CellStar, D. Dutscher, Brumath, France) and maintained in 3% DCC medium. Cells were incubated with compounds for 16 h. At the end of the incubation, effector containing medium was removed and replaced by 0.3 mM luciferin containing 6% DCC-FCS. At this concentration, luciferin diffuses into the cell and produces a stable luminescent signal. The 96-well plate was then introduced in a microplate luminometer (Microbeta, Wallac) and intact living cell luminescence measured for 2 s. EC50 values were evaluated using Graph-Pad Prism Statistics software (version 4.0; Graph-Pad Software Inc., San Diego, CA, USA).

### Western blot analysis

Proteins were resolved using a 10% SDS-PAGE. Gels were transferred to nitrocellulose using a Tris-glycine refrigerated transfert buffer, for 45 min at 100 V. Blots were incubated for 4 hr and 1 hr with the appropriate dilution of the first and second antibody, respectively. Detection was carried out using the ECL detection system according to the manufacturer's instructions.

### Ligand binding analysis

HELN-Fα/β cells were seeded at a density of 10^5 ^cells/well in 24-well tissue culture plates and grown in 6% DCC-FCS. Cells were labeled with 0.01-3 nM [^3^H]-E2 (84 Ci/mmol specific activity) at 37°C for 6 h in the absence or presence of 100 nM of non-radioactive E2. The final incubation volume was 400 μl and each dilution was performed in duplicate. After incubation, unbound material was aspirated and cells washed three times with 400 μl of cold PBS. Then, 250 μl lysis buffer (400 mM NaCl, 25 mM Tris phosphate pH 7.8, 2 mM DTT, 2 mM EDTA, 10% glycerol, 1% triton X-100) was added and plates were shaken for 5 min. Supernatant (200 μl) was mixed with 4 ml of LSC-cocktail (Emulsifier-Safe, Packard BioScience) and [^3^H] bound radioactivity was liquid scintillation counted (Packard Tri-Carb 2100TR, Perkin Elmer France). Protein concentration was determined by Bio-Rad protein assay. Specific binding was determined by subtracting nonspecific binding from total binding and free ligand concentration was estimated by subtracting total bound ligand from added ligand. The dissociation constant (Kd) value was calculated as the free concentration of radioligand at half-maximal binding by fitting data to the Hill equation and by linear Scatchard transformation.

### ChIP Assays

ChIP assays were performed as described by Metivier et al. [19] with minor modifications. Cells were cross-linked with 1.5% formaldehyde at 37°C for 5 min and resuspended in the Cell buffer (100 mM Tris-HCl, pH 9.4, 100 mM DTT). They were incubated on ice for 10 min and subsequently at 30°C for 15 min. After cell lysis and sonication, immunoclearing was performed in the presence of 5 μg of sheared salmon sperm DNA (Sigma), Immunoprecipitations were performed overnight in the presence or not of 2 μg of selected antibody. Complexes were recovered by a 2 hr incubation with protein A sepharose CL4B saturated with salmon sperm DNA. Beads were sequentially washed in buffer I (2 mM EDTA, 20 mM Tris-HCl, pH8.1, and 150 mM NaCl), buffer II (2 mM EDTA, 20 mM Tris-HCl, pH 8.1, 0.1% SDS, 1% Triton X-100, and 500 mM NaCl), buffer III (1 mM EDTA, 10 mM Tris-HCl, pH 8.1, 1% Nonidet P-40, 1% deoxycholate, and 250 mM LiCl), and three times with Tris-EDTA buffer. Washed resin was resuspended in elution buffer (1% SDS, 0.1 M NaHCO_3_) with 30-min incubation and the cross-link was reversed at 65°C overnight. DNA was purified with QIAquick columns (Qiagen, France). After immunoprecipitation with either anti FLAG, anti ERα, or anti Pol II antibodies, PCR where performed with the following oligonucleotides: pS2/*TFF1 *promoter: (S) GCCATCTCTCACTATGAATCACTT; (AS) GGGCAGGCTCTGTTTGCTTA. Luciferase transgene promoter: (S) CGACTCTAGCGGAGGACTGT; (AS) TTGGCGTCTTCCATTTTACC.

## Authors' contributions

VC and EB conceived the study. PB participated to helpful discussions and for the isolation of stable transfectants. ChIP experiments were performed by EB with help and advices from RM. Luciferase analysis experiments were performed by PB and binding assays by AE. The manuscript was prepared by EB. VC discussed analyses, interpretation and presentation. All authors have contributed to, seen and approved the manuscript

## References

[B1] Enmark E, Pelto-Huikko M, Grandien K, Lagercrantz S, Lagercrantz J, Fried G, Nordenskjold M, Gustafsson JA (1997). Human estrogen receptor beta-gene structure, chromosomal localization, and expression pattern. J Clin Endocrinol Metab.

[B2] Menasce LP, White GR, Harrison CJ, Boyle JM (1993). Localization of the estrogen receptor locus (ESR) to chromosome 6q25.1 by FISH and a simple post-FISH banding technique. Genomics.

[B3] Kuiper GG, Enmark E, Pelto-Huikko M, Nilsson S, Gustafsson JA (1996). Cloning of a novel receptor expressed in rat prostate and ovary. Proc Natl Acad Sci USA.

[B4] Zhao C, Dahlman-Wright K, Gustafsson J (2008). Estrogen receptor β: an overview and update. Nuclear Receptor Signaling.

[B5] Harris HA (2007). Estrogen receptor-beta: recent lessons from in vivo studies. Mol Endocrinol.

[B6] Koehler KF, Helguero LA, Haldosen LA, Warner M, Gustafsson JA (2005). Reflections on the discovery and significance of estrogen receptor beta. Endocr Rev.

[B7] Pettersson K, Gustafsson JA (2001). Role of estrogen receptor beta in estrogen action. Annu Rev Physiol.

[B8] Matthews J, Gustafsson JA (2003). Estrogen signaling: a subtle balance between ER alpha and ER beta. Mol Interv.

[B9] Ogawa S, Inoue S, Watanabe T, Orimo A, Hosoi T, Ouchi Y, Muramatsu M (1998). Molecular cloning and characterization of human estrogen receptor betacx: a potential inhibitor ofestrogen action in human. Nucleic Acids Res.

[B10] Vladusic EA, Hornby AE, Guerra-Vladusic FK, Lupu R (1998). Expression of estrogen receptor beta messenger RNA variant in breast cancer. Cancer Res.

[B11] Moore JT, McKee DD, Slentz-Kesler K, Moore LB, Jones SA, Horne EL, Su JL, Kliewer SA, Lehmann JM, Willson TM (1998). Cloning and characterization of human estrogen receptor beta isoforms. Biochem Biophys Res Commun.

[B12] Xu L, Pan-Hammarstrom Q, Forsti A, Hemminki K, Hammarstrom L, Labuda D, Gustafsson JA, Dahlman-Wright K (2003). Human estrogen receptor beta 548 is not a common variant in three distinct populations. Endocrinology.

[B13] Forster C, Makela S, Warri A, Kietz S, Becker D, Hultenby K, Warner M, Gustafsson JA (2002). Involvement of estrogen receptor beta in terminal differentiation of mammary gland epithelium. Proc Natl Acad Sci USA.

[B14] Liu MM, Albanese C, Anderson CM, Hilty K, Webb P, Uht RM, Price RH, Pestell RG, Kushner PJ (2002). Opposing action of estrogen receptors alpha and beta on cyclin D1 gene expression. J Biol Chem.

[B15] Metivier R, Reid G, Gannon F (2006). Transcription in four dimensions: nuclear receptor-directed initiation of gene expression. EMBO Rep.

[B16] Burakov D, Crofts LA, Chang CP, Freedman LP (2002). Reciprocal recruitment of DRIP/mediator and p160 coactivator complexes in vivo by estrogen receptor. J Biol Chem.

[B17] Shang Y, Hu X, DiRenzo J, Lazar MA, Brown M (2000). Cofactor dynamics and sufficiency in estrogen receptor-regulated transcription. Cell.

[B18] Reid G, Hubner MR, Metivier R, Brand H, Denger S, Manu D, Beaudouin J, Ellenberg J, Gannon F (2003). Cyclic, proteasome-mediated turnover of unliganded and liganded ERalpha on responsive promoters is an integral feature of estrogen signaling. Mol Cell.

[B19] Metivier R, Penot G, Hubner MR, Reid G, Brand H, Kos M, Gannon F (2003). Estrogen receptor-alpha directs ordered, cyclical, and combinatorial recruitment of cofactors on a natural target promoter. Cell.

[B20] Metivier R, Gallais R, Tiffoche C, Le Peron C, Jurkowska RZ, Carmouche RP, Ibberson D, Barath P, Demay F, Reid G (2008). Cyclical DNA methylation of a transcriptionally active promoter. Nature.

[B21] Lewandowski S, Kalita K, Kaczmarek L (2002). Estrogen receptor beta. Potential functional significance of a variety of mRNA isoforms. FEBS Lett.

[B22] Liu Y, Gao H, Marstrand TT, Strom A, Valen E, Sandelin A, Gustafsson JA, Dahlman-Wright K (2008). The genome landscape of ERalpha- and ERbeta-binding DNA regions. Proc Natl Acad Sci USA.

[B23] Matthews J, Wihlen B, Tujague M, Wan J, Strom A, Gustafsson JA (2006). Estrogen receptor (ER) beta modulates ERalpha-mediated transcriptional activation by altering the recruitment of c-Fos and c-Jun to estrogen-responsive promoters. Mol Endocrinol.

[B24] Murphy LC, Peng B, Lewis A, Davie JR, Leygue E, Kemp A, Ung K, Vendetti M, Shiu R (2005). Inducible upregulation of oestrogen receptor-beta1 affects oestrogen and tamoxifen responsiveness in MCF7 human breast cancer cells. J Mol Endocrinol.

[B25] Secreto FJ, Monroe DG, Dutta S, Ingle JN, Spelsberg TC (2007). Estrogen receptor alpha/beta isoforms, but not betacx, modulate unique patterns of gene expression and cell proliferation in Hs578T cells. J Cell Biochem.

[B26] Balaguer P, Francois F, Comunale F, Fenet H, Boussioux AM, Pons M, Nicolas JC, Casellas C (1999). Reporter cell lines to study the estrogenic effects of xenoestrogens. Sci Total Environ.

[B27] Escande A, Pillon A, Servant N, Cravedi JP, Larrea F, Muhn P, Nicolas JC, Cavailles V, Balaguer P (2006). Evaluation of ligand selectivity using reporter cell lines stably expressing estrogen receptor alpha or beta. Biochem Pharmacol.

[B28] Meyers MJ, Sun J, Carlson KE, Marriner GA, Katzenellenbogen BS, Katzenellenbogen JA (2001). Estrogen receptor-beta potency-selective ligands: structure-activity relationship studies of diarylpropionitriles and their acetylene and polar analogues. J Med Chem.

[B29] Stauffer SR, Coletta CJ, Tedesco R, Nishiguchi G, Carlson K, Sun J, Katzenellenbogen BS, Katzenellenbogen JA (2000). Pyrazole ligands: structure-affinity/activity relationships and estrogen receptor-alpha-selective agonists. J Med Chem.

[B30] George AA, Schiltz RL, Hager GL (2009). Dynamic access of the glucocorticoid receptor to response elements in chromatin. Int J Biochem Cell Biol.

[B31] Subramanian K, Jia D, Kapoor-Vazirani P, Powell DR, Collins RE, Sharma D, Peng J, Cheng X, Vertino PM (2008). Regulation of estrogen receptor alpha by the SET7 lysine methyltransferase. Mol Cell.

[B32] Kangaspeska S, Stride B, Metivier R, Polycarpou-Schwarz M, Ibberson D, Carmouche RP, Benes V, Gannon F, Reid G (2008). Transient cyclical methylation of promoter DNA. Nature.

[B33] Hu P, Kinyamu KH, Wang L, Martin J, Archer TK, Teng CT (2008). Estrogen induces estrogen-related receptor alpha gene expression and chromatin structural changes in estrogen receptor (ER)-positive and ER-negative breast cancer cells. Biol Chem.

[B34] Lonard DM, Nawaz Z, Smith CL, O'Malley BW (2000). The 26S proteasome is required for estrogen receptor-alpha and coactivator turnover and for efficient estrogen receptor-alpha transactivation. Mol Cell.

[B35] Fowler AM, Solodin NM, Valley CC, Alarid ET (2006). Altered target gene regulation controlled by estrogen receptor-alpha concentration. Mol Endocrinol.

[B36] Park S, Song J, Joe CO, Shin I (2008). Akt stabilizes estrogen receptor alpha with the concomitant reduction in its transcriptional activity. Cell Signal.

[B37] Duong V, Boulle N, Daujat S, Chauvet J, Bonnet S, Neel H, Cavailles V (2007). Differential regulation of estrogen receptor alpha turnover and transactivation by Mdm2 and stress-inducing agents. Cancer Res.

[B38] Zhao C, Matthews J, Tujague M, Wan J, Strom A, Toresson G, Lam EW, Cheng G, Gustafsson JA, Dahlman-Wright K (2007). Estrogen receptor beta2 negatively regulates the transactivation of estrogen receptor alpha in human breast cancer cells. Cancer Res.

[B39] Hodges-Gallagher L, Valentine CD, El Bader S, Kushner PJ (2008). Estrogen receptor beta increases the efficacy of antiestrogens by effects on apoptosis and cell cycling in breast cancer cells. Breast Cancer Res Treat.

